# Effective interventions to improve the health literacy of cancer patients

**DOI:** 10.3332/ecancer.2019.966

**Published:** 2019-10-10

**Authors:** Loreto Fernández-González, Paulina Bravo-Valenzuela

**Affiliations:** 1 Dalla Lana School of Public Health, University of Toronto, 155 College Street, Toronto, ON M5T 3M7, Canada; 2 Nursing School, Pontificia Universidad Catolica de Chile, Santiago 7820436, Chile; 3 School of Social Sciences, Cardiff University, Cardiff CF10 3NN, UK

**Keywords:** health literacy (HL), health education, cancer, result evaluation (health care), literature review

## Abstract

Health literacy (HL) refers to the cognitive and social abilities that are determinants in the motivation and capacity of the individual to access, understand and use information for the care of one’s own health. In oncology, increased survival, navigation of the healthcare system, the many different forms of treatment and the management of adverse effects/outcomes make HL a critical factor in patient care. The objective of this study is to identify the structure, content and effectiveness of interventions to improve HL in cancer patients.

**Materials and methods:**

A literature review was performed using the ‘(health literacy OR Cancer Literacy) AND Cancer AND Intervention’ strategy on seven multidisciplinary databases. Studies that intervened in subjects diagnosed with cancer and treating HL explicitly as a variable to be measured were included.

**Results:**

One thousand two hundred and thirty-six abstracts were retrieved. Eight studies met the inclusion criteria. Research focused on patients diagnosed with breast cancer or prostate cancer. Interventions used multimedia resources and face-to-face interactions. No study defined HL. HL was usually a secondary outcome. There is high variability in the design of studies and interventions and in the instruments used to measure HL. The effectiveness of the interventions varied between studies, with improvements that were diminished over time or insufficient in participants with initial low literacy.

**Conclusion:**

The evidence to date in interventions oriented to study HL in patients with cancer is focused on other constructs, leaving HL as a phenomenon difficult to define both conceptually and clinically. Variability in designs and measurements makes comparison between interventions difficult. Defining and operationalizing HL is critical to design and measure effective interventions, which must be adapted to patients’ needs.

## Introduction

Health literacy (HL), also known as functional HL, refers to the cognitive and social abilities that have a determining role in the motivation and capacity of the individual to access, understand and use information in order to promote their own health [1]. The concept of HL was introduced in the mid-seventies and has been gaining relevancy to the extent that evidence shows that it is the strongest predictor of a person’s health status, along with other social determinants such as age, income, work status, educational level and race/ethnicity [2]. For example, those subjects and groups with low levels of HL are at risk of having worse health than those who possess better HL, which leads to the reproduction of health inequities [1]. According to Edwards *et al* [3], the conceptual development of HL has been evolving from a notion fundamentally centred on the cognitive ability of the patient to adequately process information to models based on action orientation and successful interactions with healthcare providers and participation in the decision-making process. There are currently multiple definitions and conceptual models to understand HL. The model presented by the World Health Organisation (WHO) [1] based on the *European Health Literacy Consortium* includes 12 subdivisions under the categories of health promotion, illness prevention and healthcare. From this point of view, HL integrates and interacts with concepts such as empowerment and sustainability. Developing a HL model for chronic illnesses, Edwards *et al* [3] distinguished eight elements: health knowledge, self-care skills, active search and use of information, active communication with the healthcare team, search of treatment options, decision-making, influence of others in HL and expected results in HL.

Defining HL is key to operationalize and measure the construct in terms of low or high HL, and for designing interventions aiming to improve it. This is not only critical from a scientific and methodological point of view but also from the point of view of public policy for implementing evidence-based strategies to promote HL [4, 5].

This fact is particularly important from a public health viewpoint, given the sustained increase in chronic illnesses that require constant effort in self-management by patients and their caregivers [3]. On the other hand, the complexity of health systems and the development of new communication technologies have allowed immediate and continued access to health information, entailing a challenge to individuals, governments and international organisations [2], which can impact people’s level of HL. In addition, research has demonstrated that the subjects with low HL have limited participation in healthcare appointments and in decision-making, make more calls to emergency services, have a higher number of hospitalisations and present poor self-care in general [3], thereby directly impacting healthcare costs.

HL is an area of health promotion in terms of the adoption of healthy habits and early detection behaviours, particularly necessary in patients with chronic illnesses, which have increased globally both in incidence and survival [3, 6]. Daily management of chronic illness requires the use of functional, cognitive and social resources. Optimal care for these patients involves frequent contact with the healthcare system and active management of the health condition. People with low HL engage less effectively in activities related to their own health management, resulting in increased disease burden and overall worse health outcomes [6, 7]. Therefore, an adequate and comprehensive understanding of patients’ HL is necessary if successful interventions and services are intended to be implemented. In the context of cancer care, the increase in survival, the navigation in the healthcare system, the multiplicity of treatment options and the management of adverse effects/sequelae of short, medium and long terms make HL a critical factor in patient care [8].

Regarding conceptual models on HL in cancer and its role in health outcomes, the work of Echeverri *et al* [9] stands out; they define it as the ability of the individual to search, understand, evaluate and use basic information to take appropriate decisions regarding prevention, diagnosis and treatment of cancer. Due to the fact that it is not directly observable and has a multidimensional nature [9], the development of interventions designed to improve HL in cancer poses important methodological and technical challenges.

Knowing the type of interventions—both clinical and community-based—and their effectiveness described in the literature constitutes a logical and decisive step in order to implement and tailor them to local contexts. Hence, the present article is a structured literature review conducted with the objective of knowing and describing the effectiveness of interventions aimed at improving the HL of patients diagnosed with cancer.

## Materials and methods

A structured review of available evidence was conducted following international recommendations for the assurance of methodological rigour [10, 11]. Due to the exploratory nature of the review, a narrative review was conducted rather than a systematic one, focused on qualitatively describing the studies and their strengths and weaknesses, following PRISMA guidelines in the screening and selection of the articles to ensure the inclusion of relevant studies. For this reason, we used the term ‘intervention’, understanding this as the development of actions regarding the condition to modify the cancer HL of the participants, whether by the increase in knowledge, self-care strategies, decision-making to the condition, use of health services or similar. In the same way, no *a priori* definitions or specific conceptual frameworks of HL were used to restrictively select the studies.

### Search strategy

The inclusion criteria for the articles were: 1) studies published in scientific journals in English or Spanish with no start date limit until 2017; 2) studies reporting interventions that explicitly consider HL as an outcome or variable and 3) the participants of the study are cancer patients with any type of cancer and stage. Studies centred on the development of instruments for measuring HL, or conducted in healthy or non-oncological population were excluded.

With the goal of identifying pertinent articles, the following search algorithm was constructed with the help of an expert librarian: ‘(Health* Literacy* OR *Cancer Literacy*) AND *Cancer* AND *Intervention*’ in ‘All categories’ (or its nearest equivalent according to the database used), in English and Spanish. The following databases were selected, privileging their multidisciplinary nature in health: *Pubmed, Web of Science, CINAHL, PsycInfo, Scopus, Epistemonikos* and* Scielo*. The literature search in databases was conducted during the month of May 2017.

### Studies selection and data extraction

Types of texts were not filtered* a priori* to cover as many documents as possible. Articles of qualitative, quantitative or mixed methodology were included. The systematic or narrative reviews were included as a source of possible articles of interest in their references. Grey literature was not considered. Once duplicates were eliminated, a researcher (LFG) reviewed the titles of all the articles, verifying compliance with the inclusion criteria. An independent screening was conducted on 10% of the titles between both authors (LFG and PBV) in order to unify selection criteria. The following phase included the reading of the abstracts in order to evaluate the pertinence of the study’s inclusion in accordance with the objective of this review. A narrative analysis was conducted on the information obtained, categorising the characteristics of the interventions and the report of their effectiveness [12]. Data extraction was conducted using a matrix including general information (year and country), type of study, type of cancer and sample data, definition of HL used, characteristics of the intervention, instruments used for measuring HL, the effects of the intervention and results obtained.

**Figure 1.**
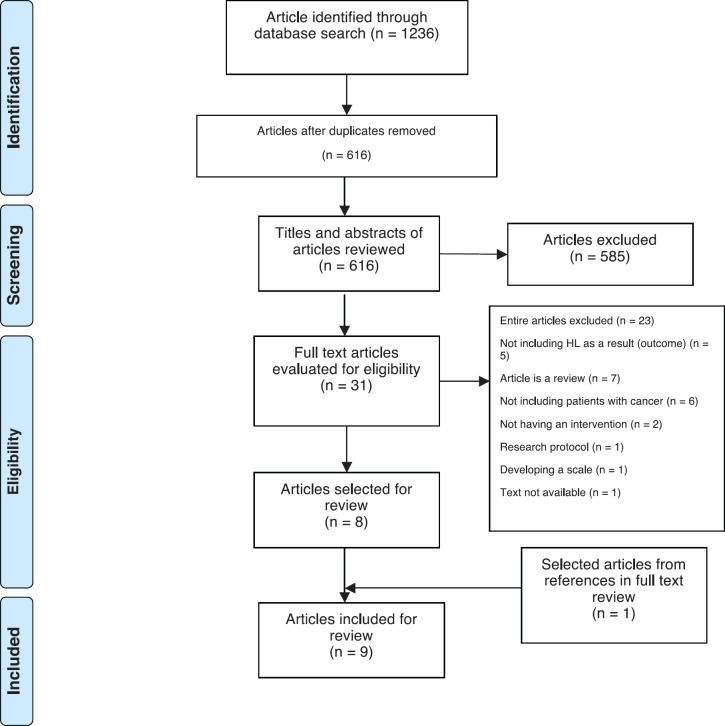
Flowchart of the article identification process.

1,236 results were retrieved. Duplicates were eliminated, resulting in 616 articles that were reviewed in title and abstract. From this phase, articles for full reading were selected, of which one article was excluded due to the unavailability of the original article. In addition, in this phase of exploratory screening, one additional article from a systematic review that met the inclusion criteria was included. Thirty-one articles were selected for full-text reading for further evaluation. Of these, 23 articles were discarded, either for not reporting HL in their results (*N* = 5) for corresponding to a literature review (*N* = 7), for being articles whose studies do not include subjects with cancer (*N* = 6) or do not include an intervention (*N* = 2), for corresponding to a research protocol (*N* = 1) and finally, for reporting the development of a scale and not a intervention (*N* = 1). Finally, a total of nine articles corresponding to eight studies (one study published preliminary and final results, both were included) met the inclusion criteria were included in the review. The selection process of the articles is shown in Figure 1.

## Results

Table 1 summarises the characteristics of the articles that met the inclusion criteria.

### Characteristics of the studies

All studies included according to the exposed criteria were conducted in the United States. Regarding the type of cancer diagnosis, all studies worked with adult patients with only one type of cancer, with 50% (4/8) of the studies conducted in women patients with breast cancer [14–18] and 50% (4/8) in patients with prostate cancer [19–22]. Only one study [17] included both healthy subjects and diagnosed patients. Only one study included solely patients with non-metastatic cancer [14, 15], while 3/8 reported including subjects with early and advanced stages of cancer [18, 19, 22]; in other studies, these data were not reported. The types of studies selected utilised designs with qualitative (*N* = 1) [16] and quantitative methods such as randomised clinical trials (*N* = 3) [12, 13, 15, 19] and prospective cohort studies (*N* = 4) [18, 20, 22]. The sample size of the studies has an average of 71 patients, varying between 12 [16] and 130 patients [18], with an emphasis on the participation of minorities in the American context (African–American and Latino/as). The outcomes of the studies include increased knowledge regarding one’s own health [18, 22], decision-making regarding oncological treatments [14, 15, 19], management of specific tumour site terminology [20], improving adherence to medical treatments [17, 18], adoption of self-care practices [15, 19] and promoting access to participation in clinical trials [16]. The tools used in the studies show great variability, with the questionnaire *Rapid Estimate of Adult Literacy in Medicine* (REALM) being the most used, in 37.5% of the studies (3/8) [19–21]. Regarding HL conceptualization, none of the studies report a definition of the construct.

### Characteristics of the interventions

The interventions conducted vary in content as well as the mode of delivery. The use of audiovisual materials is featured in 50% of the studies, where the contents are transmitted mainly through videos created especially for the intervention. These videos may contain a narrative approach [14, 15], or mostly illustrative on concepts related to organs affected by cancer according to the tumour site, information regarding treatments and management of adverse effects of treatments [19–21]. In addition to the use of videos, interviews are conducted and/or printed materials are provided with the goal of contextualising the specific clinical situation to each patient [14, 15, 19, 21]. Other interventions opt to provide written material in card format [16, 18], whose content is personalised according to the participant’s health status, with relevant information according to the objective of the study. Individual and personalised work format is privileged, with only one of the interventions performed in the group workshop format [17]. As for the professionals or roles of those who deliver the interventions, the participants were predominantly nurses [21, 22], with minor participation of social workers [17], pharmacists [17] community health workers [18], and figures such as case managers [14, 15] or navigators [16, 18].

### Effectiveness of interventions

The effectiveness of the intervention was only reported in those clinical trials that included a control group [14, 15, 17, 21]. For those studies designed with pre- and post-intervention measurements without a control group, the variation in the variable of interest or expected result is reported. For its part, the only qualitative study participants was centred on reporting information of the acceptability and experience of the intervention by the study participants and those that provide the intervention [16]. In general, studies that reported acceptability or conformity of interventions show high satisfaction with the intervention and its content [16, 18, 19]. Furthermore, there is usually a correlation between reported levels of HL and other variables, such as the use of self-care techniques [21], knowledge regarding the illness [19], self-efficacy [17] or adherence to treatments [17].

Clinical trials that include a control group present diverse results. While Rust *et al* [17] reported an absence of significant differences between both groups after the intervention (*p* = 0.83), Jibaja-Weiss *et al* [15] observed that the intervened patients, contrary to the study’s hypothesis, tended to choose complete mastectomies over conservative surgeries despite having a significant increase in the understanding of the disease and its treatment options. In addition, one year follow-up shows no difference between groups regarding knowledge and decisional conflict. On the other hand, in a study by Wilson *et al* [21] the number of adverse effects to radiation therapy was similar between groups and between patients with high versus low literacy. At the same time, it is observed that the average use of self-care techniques increases over time in all groups, independent of the level of HL, except in the subgroup of patients with low HL belonging to the control group, in which it decreases.

Prospective cohort studies also present mixed results. The study by Kim *et al* [19] consists of an audiovisual intervention through compact discs (CD ROM) with the goal to assist in the decision-making process, and had high acceptability, with two-thirds of the patients being able to make a decision by the end of the intervention. However, half of these patients were later prescribed a different treatment decided by themselves during the study. In addition, a correlation between low HL and limited knowledge about prostate cancer was reported. Ulloa *et al* [18] also reported a high acceptability of the intervention (personalised cards) and an increase in disease knowledge and health status of the participating patients. As for Wang *et al* [20], the results show an increase in the understanding of prostate cancer terminology, with a significant improvement in patients with low HL in the understanding of terminology related to sexual function compared to subjects with high HL. Finally, Zavala *et al* [22] reported an increase in accurate self-reporting of prostate antigen (PSA) during participant follow-up.

## Discussion

The present paper reviews a series of interventions conducted in cancer patients intending to improve their HL. The studies that met these criteria present similarities and differences. Among the similarities, all the studies were conducted in the United States with an important emphasis on including groups considered vulnerable such as Latinos and African Americans. This is consistent with one of the principles that support the relevance of the concept of HL, which is to improve the health outcomes of at-risk groups in order to overcome socio-health inequities [1]. Among the most salient differences is the variability in the methodological design, the content of the interventions and their results.

All studies were designed for subjects with the same cancer diagnosis, either breast or prostate cancer. This implies that the interventions include: 1) specific information about this type of cancer and 2) took certain elements either in their format or content that allude to the diverse gender attitudes present in social discourse, with the goal of achieving closeness and improving acceptability of the intervention. Thus, for example, Jibaja-Weiss *et al* [14, 15] developed an audiovisual format that emulated a soap opera, while the rewards for patients who reached self-care goals in Wilson’s study included male personal care products or tickets for sporting events [21]. The fact that the interventions encompass subjects with the same type of tumour also allowed them to focus on specific aspects of these cancers, such as the self-reporting of PSA [22] or the enrolment in certain clinical trials [14]. The choice of these types of cancer is consistent with both the respective prevalence and the increased survival of the patients [23], as with the trends of prioritisation in cancer research [24]. It is expected that the future research will aim to design interventions targeting subjects with other neoplasms in which HL plays a critical role [25].

The fact that none of the articles reported a definition of HL makes its operationalisation difficult in terms of research and also its conceptual differentiation from other related concepts and variables. A routine question arises as to whether HL is really an isolable object of study, or if it is rather an epiphenomenon of certain situations that occur in health contexts. The difficulty in theoretically isolating the concept and the consensus over its definition has been a constant source of debate between specialists in the field [24]. This difficulty is even greater if one takes into account the multidimensionality of the concept, its relative recent appearance in the scientific field and the variety of instruments that have been developed to date, which emphasize different dimensions of the concept depending on the definition used when designing the tool [3, 7, 27, 28]. Consensus on this conceptualisation is of vital importance for the design and reporting of the effectiveness of interventions that promote HL, as has happened in the development of other constructs such as health empowerment [29].

Accordingly, it is observed that the totality of the studies did not seek improvement of HL as a primary outcome but instead seek some health behaviour or the increase in the knowledge related to the oncological diagnosis. From these studies, it follows that HL is a variable that correlates with observable behaviours, or measurable outcomes, rather than an outcome itself. This is crucial from a research perspective since it constitutes a key element in the effective evaluation of the interventions performed. In the studies reviewed in this paper, it is observed that the logic of the interventions performed is consistent with the conceptual framework of Echeverri *et al* [9]. The interventions aimed primarily at improving either the knowledge about the illness or the respective treatments [14, 15, 18–20], as well as self-knowledge of one’s own health status [18, 21, 22] in order to carry out actions related to decision-making about treatments [14, 15, 19], generate adherence and self-care practices [17, 19], and participation in research [16]. However, the poor conceptualisation of the construct and the use of questioned instruments for the measurement of HL [30] are problematic since they raise the question of what is understood by HL and, more importantly, how to modify it effectively.

In effect, it is important to clearly define if the effectiveness of the intervention will be given by the observable changes in the primary outcome or if an intervention that produces results different from those expected *while* increases elements that are considered part of HL will also be considered as effective. The study conducted by Jibaja-Weiss *et al* [15] is illustrative of the dilemma: the patients increased their knowledge of breast cancer as expected, but their attitudes about the treatment of choice changed contrary to the hypothesis, opting more for a different treatment than expected. The study carried out by these authors, therefore, reveals the complexity of HL and its multidimensionality, where behaviours related to treatments associated with greater HL do not necessarily derive linearly from handling more information about cancer. In this way, it is observed that the behaviours related to HL are determined by multiple components where managing information or having knowledge about the health condition is not synonymous with making decisions based on scientific evidence.

The poor conceptualization of HL in the articles was the greatest finding, as well as the greatest challenge, of this paper. When grouping the studies and their characteristics, although the included studies met the inclusion criteria of explicitly reporting HL as a result, in the end, a detailed review of the articles found that they were studies whose focus was primarily other constructs that were affected by the multidimensionality of HL in their dimensions of knowledge, attitudes, actions and decisions related to cancer behaviours [9]. This makes it impossible to compare the studies, impeding the extraction of conclusions regarding the most effective intervention strategies for vulnerable groups with low HL.

Although no similar reviews were found for the association between HL and interventions for cancer patients, this literature review is consistent with reviews of HL and other health conditions [5, 31–35]. In general, there is consensus on the need to improve the methodological quality of the designs, and the reporting of results of the interventions, especially in the long term, in order to find more robust associations between HL and health outcomes, emphasising the development of interventions for vulnerable groups and their participation in studies. The work of Brainard *et al* [5], specifically oriented to the methodological quality of clinical trials in HL, highlights the importance of focusing interventions on patient-centred outcomes, in terms of developing high-quality evidence, with meaningful clinical implications. On the other hand, the review conducted by Geboers *et al* [32] focused on HL and adherence in older adults, underscoring the variability of the interventions and their quality, which makes it impossible to draw conclusions on the most effective type of intervention in this field. Sheridan *et al* [34] suggested that although certain interventions for people with low HL have been shown to be effective, it is important to continue developing new formats and piloting interventions that combine different elements that can adapt both to the groups they are targeting and to the health condition and its consequences they are seeking to mitigate. Their conclusions are consistent with the results of this review regarding the correlation between educational level and HL level in study participants. Certainly, those subjects with lower educational level and low HL are the ones who can benefit mostly from interventions aimed at improving HL, but this requires the use of specific strategies that favour the use of animations, videos or other devices that do not require advanced levels of literacy [9]. However, despite both methodological and theoretical difficulties, the authors agree that HL is a promising field of research and development, with a potential impact on patients and health services that merits further study.

One of the strengths of this review is that, to our knowledge, this is the first review carried out on this topic in Spanish (see Spanish version pdf), which accounts for the novelty of the topic and its dissemination in our language. This is a structured review of the literature, so the steps proposed by PRISMA were followed to ensure the quality of the process. Although the articles included were published in journals with a peer review process, and the studies were approved by scientific ethics committees for the protection of the participants, a limitation is that not all the steps established in the PRISMA checklist were performed.

This review could guide health teams in oncology for the development and implementation of interventions at different levels of health services that promote HL, an element that could be considered good clinical practice that entails not only people knowing and understanding more about their disease and treatment options, but also respecting the autonomy and dignity of patients [37]. According to the results, it is feasible to suggest that future research on the subject should be aimed at strengthening the link between theoretical understanding of HL, its measurement and operationalisation and the design of methodologically rigorous studies. In addition, it is appropriate to propose that the acceptability of the intervention should be a reported outcome in a standardised manner in all studies, both by the participating patients and those who deliver the intervention [33]. Finally, it is expected that soon there will be evidence from other countries and cultural realities, as well as the development of interventions and protocols that include other types and stages of cancer (for example, advanced cancer or in the remission/follow-up stage).

## Conclusion

The promotion of HL in cancer patients is a necessary strategy for delivering quality and patient-centred care. The current state of evidence on the subject accounts for an incipient area of research, is focused mainly on the United States and on breast or prostate cancer. Despite this, the research conducted is heterogeneous in terms of methodological design and the role given to HL in the intervention. The evidence to date aimed at intervening HL in patients with cancer focuses on other constructs, leaving HL as a phenomenon difficult to define conceptually and clinically.

However, it is possible to extract what elements common to the interventions are: 1) the combination of face-to-face interactions with health professionals and the use of multimedia technologies or devices, 2) the emphasis on identifying vulnerable individuals and groups with low HL and 3) the notion that HL does not operate in isolation but is directly associated with education level, behaviours and factors that influence decisions about one’s own health.

Accordingly, the first step for the successful development of HL-related research in oncology is the rigorous and consensual conceptualisation of HL, as well as its delimitation and relationship with other clinical and psychosocial factors that affect the participation and well-being of the person who is being treated for cancer. The progress in research in the area will, in turn, perfect the design of effective interventions, adjust the format and choose appropriate instruments to measure the impact of interventions aimed at improving the impact of HL in the prevention, diagnosis and treatment of cancer according to the characteristics of each patient group.

## Conflicts of interest

The authors declare that they have no conflicts of interest.

## Funding declaration

This work was supported by the CONICYT National Commission for Scientific and Technological Research National Fund for Scientific and Technological Development (FONDECYT) Chile, FONDECYT Project Initiation in research grant no. 1115221. FONDECYT had no influence over the design of the study; in the collection, analysis or interpretation of the data, or in the preparation, review or approval of the manuscript.

## Figures and Tables

**Table 1.** Articles included in the review.

Article	Type of study	Country of study	Sample	Result or variable of interest (outcomes)	HL measurement and other instruments used	Intervention characteristics	Results obtained
Jibaja-Weiss *et al* [14]	Randomised controlled trial. Only intervened group is reported.	US	Fifty-one women with breast cancer recently diagnosed. Majority of Latin or African American origin.	Decision-making regarding breast surgery.	1) Decisional conflict scale (low literacy version).	(CPtDA): computerised patient decision aid. Edutainment (a patchwork of life: a woman’s story for making breast cancer treatment decisions). Video with soap opera narrative structure and interactive learning modules. Patient watches the video after medical consultation and receives personalised brochure.	There is a significant decrease in uncertainty and personal values after the intervention.
Jibaja-Weiss *et al* [15]	Randomised controlled trial	US	One hundred women recently diagnosed with breast cancer. Majority of Latin or African American origin.	Decision-making regarding breast surgery.	1) Decisional conflict scale (low literacy version). 2) Breast cancer knowledge questionnaire. 3) Satisfaction with decision scale. 4) Satisfaction with the decision-making process questionnaire. 5) Satisfaction with programme questionnaire.	CPtDA. Edutainment (a patchwork of life: a woman’s story for making breast cancer treatment decisions). Video with soap opera narrative structure and interactive learning modules. Patient watches the video after receiving medical consultation and personalised brochure.	Unexpected result: intervened group chooses more to have modified radical mastectomy. There is a significant difference between groups in knowledge about the disease, but they are equal after one year. There is no significant difference between groups in satisfaction with decision. The decision conflict decreases over time, with both groups equalising each year.
Kim *et al* [19]	Prospective cohort	US	Thirty patients recently diagnosed with prostate cancer. Half African Americans.	Shared decision-making regarding treatments for prostate cancer.	1) Prostate cancer knowledge questionnaire (PCKQ). 2) Rapid estimate of adult literacy in medicine (REALM).	CD-ROM with shared decision-making programme. Includes videos, images and personalised information about the disease. Follow-up to check treatment choice and treatment performed.	More than 75% of the patients rated the programme as very satisfactory. Patients’ HL correlates with their level of knowledge about prostate cancer. Two-thirds of the patients chose a treatment option. However, one-third received a different treatment than preferred after the intervention.
Nickell et al [16]	Participatory research and intervention piloting	US	Twelve breast cancer survivors of diverse ethnicity and four clinical trial navigators.	Promote access to participation in clinical trials.	Not measured directly.	HREI: health research engagement intervention. Intervention given by personal navigator with five components: 1) explanation about health research and its value; 2) a card with health research resources, which contains a list of services with available information; 3) brief questionnaire about the medical history to identify appropriate studies; 4) personalised list with appropriate studies 5) comments on the studies.	The total number of women who responded to the follow-up (*n* = 11) showed acceptability of the intervention and of the patient navigator.
Rust *et al* [17]	Pilot randomised controlled trial	US	Forty-eight African-American breast cancer survivors.	Improvement of adherence to medical treatments.	1) Self-efficacy for appropriate medication use scale (SEAMS). 2) Adherence to refills and medications scale (ARMS). 3) Self-created questions to measure HL.	MST: The medication adherence skills training; 2-hour group workshop taught by pharmacists and social workers. The workshop content includes information on the correct use of medications, communication skills and overcoming barriers to pharmacological adherence.	There is a significant correlation between HL, self-efficacy and adherence to treatments. There are no significant differences between both groups after the intervention.
Ulloa *et al* [18]	Prospective cohort	US	One hundred and thirty women with breast cancer. Most Latin.	1) Increased knowledge about one’s own health. 2) Improvement of adherence to medical treatments. 3) Adoption of self-care practices.	Single item literacy screener. Pre-post assessment. Satisfaction with the intervention, comprehensibility of the material and communication with the healthcare team was measured.	Patient receives a card that contains information about the stage of the tumour, nodal status and treatments received. It is completed by the study coordinator, along with the delivery of recommendations for healthy living and follow-up (5 min). Then, the information is reviewed individually with the patient by a professional navigator or community health worker (10–15 min).	Knowledge about the disease (stage, treatments received, nodal status, follow-up, recommendations and healthy lifestyles) increased significantly. The card format for the wallet had high acceptability (93%).
Wang DS [20]	Prospective cohort	US	Fifty-six adult men with 10% of men diagnosed with prostate cancer. Most African Americans.	Management of specific terminology of the genitourinary system and prostate cancer.	1) Kilbridge questionnaire. 2) Schwartz-Woloshin numbering test. 3) REALM.	PCLA: prostate cancer literacy application. Audiovisual educational tool (video). Content illustrates medical terms in a clinical context. Each term is illustrated in three-dimensional animations, with some explained in greater detail in two dimensions. The use of medical jargon was avoided. The videos have a duration of 16 min and cover the urinary, sexual and intestinal systems. After each video, the patient evaluates their understanding of the contents.	There was a significant increase in the understanding of medical terminology in 13 of the 32 terms, and in the identification and location of organs of the genitourinary system. Patients with low literacy had a greater increase in the understanding of terminology related to sexual functions than subjects with high literacy; however, they had no significant differences in the understanding of urinary and intestinal terms.
Wilson *et al* [21]	Randomised three-arm controlled trial.	US	Seventy patients with prostate cancer with indication of radiotherapy. Most African Americans.	Adoption of self-care practices.	1) REALM. 2) Side-effect interview (SEI). 3) Mood’s self-care management techniques checklist.	Group 1: educational intervention arm with three videos and printed material: Patients see before the start of treatment a first video about introduction to radiotherapy treatment. Content includes the planning and treatment process, staff roles, operation of radiotherapy and treatment machines, and effects of radiotherapy on lifestyle. The second video is shown in the third week of treatment and deals with adverse effects and self-care measures. The third and final video is shown at the end of the treatment and addresses emotional reactions and ways of coping with physical and emotional effects after treatment, as well as information on resources in the community. Group 2: educational intervention arm and agreement (contract) on self-care behaviours. This strategy is implemented through a contract made between the nurse of the study and the patient according to objectives defined together in the context of treatment. This technique helps patients identify self-care behaviours regarding the physical, emotional and social adverse effects of the treatment. A care plan signed by both is established. Rewards (basic care products, etc.) are delivered upon meeting the objectives. Group 3: usual care.	Men with low literacy increased their care in the intervened groups. Adverse effects were similar in all three groups. There was an increase in the use of self-care techniques over time, but no significant differences were found between groups. The use of self-care techniques had significant differences between patients with high and low literacy.
Zavala *et al* [22]	Prospective cohort	US	One hundred and fourteen homeless men in treatment for prostate cancer. Mostly of Latin heritage.	Increased knowledge about one’s own health.	Self-report of prostate antigen (PSA).	Initial interview in which clinical and psychosocial history is collected, including PSA values. Prostate cancer education, care coordination assistance and identification of needs and available resources are carried out. Routine contacts are maintained where PSA education is reinforced, symptoms are evaluated and adherence to medical controls is monitored.	There was a growing trend in the correct report of the prostatic antigen over time (18 months). The difference was significant between patients receiving treatment in private versus public centres.
